# Determining the frequency of *Helicobacter pylori* in children with chronic kidney failure and its relationship to gastrointestinal symptoms

**DOI:** 10.1002/ccr3.9157

**Published:** 2024-07-03

**Authors:** Behgol Nemati Nezhad, Bahar Allahverdi, Farzaneh Motamed, Shirin Djalalinia, Fahimeh Askarian, Daryoush Fahimi, Behnaz Bazargani, Arash Abbasi, Mastaneh Moghtaderi

**Affiliations:** ^1^ Resident of Pediatric Course, Children Medical Center Hospital Tehran University of Medical Sciences Tehran Iran; ^2^ Pediatric Gastroenterology and Hepatology Research Center, Children's Medical Center, The Pediatric Center of Excellence Tehran University of Medical Sciences Tehran Iran; ^3^ Pediatric GI Research Center, Children's Medical Center Tehran University of Medical Sciences Tehran Iran; ^4^ Deputy of Research and Technology Ministry of Health and Medical Education Tehran Iran; ^5^ Pediatric Chronic Kidney Diseases Research Center, Children's Medical Center Tehran University of Medical Sciences Tehran Iran

**Keywords:** children, chronic kidney disease, functional dyspepsia, gastritis, *Helicobacter pylori*, peptic ulcer

## Abstract

**Key Clinical Message:**

As there is no significant mutual relationship between *Helicobacter pylori* infection and chronic kidney disease in children, its routine study is not justified and is recommended only in symptomatic cases.

**Abstract:**

Children suffering from chronic kidney disease (CKD) often complain of indigestion but, if it is accompanied by abdominal pain, it is necessary to investigate and rule out Helicobacter pylori infection to confirm functional dyspepsia. Epidemiological studies in adults have conflicting results regarding the association between *Helicobacter pylori* infection and CKD. In this study, we determined the prevalence of *H. pylori* in children with kidney failure and its relationship to their gastrointestinal symptoms. In this retrospective study, 54 children with chronic kidney failure admitted to the hemodialysis ward of the Children's Medical Center, Tehran, Iran between 2012 and 2020 were studied. The mean age of our patients was 11.89 ± 3.99 years and their sex distribution was equal. *H. pylori* infection was reported in only three patients with 5.6%. Based on our findings, epigastric pain in children was the most common gastrointestinal symptom (70.4%). Among all patients, three patients (5.6%) died, all of them were male (P = 0.075). The most prevalent underlying cause of kidney failure in our patients was neurogenic bladder. We did not find any significant relationship between the increased risk of chronic kidney failure and co‐infection with *H. pylori*. Investigating the cause of epigastric pain and looking for *H. pylori* is very important in CKD children under hemodialysis because if they receive a transplant the possibility of gastrointestinal complications will be increased with the use of steroid and immunosuppressive drugs.

## 1 INTRODUCTION

Chronic kidney disease (CKD) is defined as an irreversible reduction in kidney function, which can be fatal in the absence of dialysis or transplantation. This condition is associated with diverse clinicopathological conditions of the digestive, cardiac, vascular, pulmonary, and immune systems.^1^, ^2^ There have been discussions about the mutual relation between *Helicobacter pylori* infection and CKD.^3^, ^4^ Yet, the pathogenesis of *H. pylori* infection in CKD patients is not clearly defined.^5^, ^6^ Patients with CKD often complain of dyspepsia due to various causes, and *H. pylori* infection should be excluded from the diagnosis of functional dyspepsia.^7^

*H. pylori* is the most prevalent chronic bacterial infection in humans and is associated with several other gastrointestinal disorders including gastritis, peptic ulcer, gastric cancer, and extra‐nodal marginal zone lymphoma of mucosa‐associated lymphoid tissue.^6^, ^8^ Although *H. pylori* is observed in 20%–40% of the general population, it has been accepted as an etiological factor in peptic ulcers, chronic gastritis, indigestion, gastric, and mucosa‐related cancers.^9^ Overall, the risk of gastrointestinal complications and cancer is higher in CKD patients than in the general population.^10^ It is known that *H. pylori* can play an important role in the gastrointestinal symptoms of patients with chronic kidney failure. Also, early diagnosis and treatment for *H. pylori* infection might prevent its impact on renal function.^11^ This study is designed to investigate the prevalence of *H. pylori* in pediatric CKD and its relation to gastrointestinal symptoms.

## 2 METHOD AND MATERIALS

In this retrospective study, 54 patients with chronic kidney failure admitted to the hemodialysis ward of the Children's Medical Center, Tehran, Iran between 2012 and 2020 were studied. The sampling method was based on patients aged (1–18 years) with chronic kidney failure who were undergoing hemodialysis permanently at least once a week. Patients with the use of non‐steroidal anti‐inflammatory drugs (NSAIDs), patients with a history of *H. pylori* eradication treatment, and subjects with incomplete medical records were excluded from the study. Patients with clinical symptoms and gastrointestinal complications with a positive test were subjected to a serology test with 5 cc of blood to check IgG and IgM for *H. pylori*. Then the patients were subjected to endoscopic and serological gastrointestinal examinations. All patients underwent endoscopic study and if there were positive findings stool examinations were done to ascertain the presence of *H. pylori* antigen.

Detailed clinical data were retrospectively collected from the patient's medical records. This information included demographic characteristics, gastrointestinal symptoms such as heartburn, abdominal pain, type of drug used, method of checking *H. pylori* infection, kind of underlying disease, type of dialysis, life status, endoscopy, and pathology results. This study was approved by the Ethics Committee of Tehran University of Medical Sciences (Ethics number: IR.TUMS.CHMC.REC.1401.091).

### 2.1 Statistical analysis

Statistical analysis was done by SPSS software version 26. The qualitative variables were indicated as numbers and percentages. Analytical analysis was performed using chi‐squared test. A p < 0.05 was considered significant.

## 3 RESULTS

In this study, 54 patients with chronic kidney failure undergoing hemodialysis with gastrointestinal complaints were studied. The sex difference was equal (27 boys and 27 girls). The age range of patients was 1–18 years old, and the mean age was 11.89 ± 3.99 years. Considering the distribution of the age categories; most of the patients were in the range of 10–14 years old (*n*: 22; 40.7%).

The most common gastrointestinal symptoms in these children were epigastric pain (70.4%) and nausea (16.7%). Four (7.4%) had no signs and symptoms at all. The most commonly used drug was Famotidine (57.4%), followed by Protein Pump Inhibitor (13%), and the most combination treatments were Famotidine and PPI (*n*: 13, 24.1%). The average duration of treatment was 6.17 ± 5.03 months (range; 1–24 months). Of the entire cohort, a total of three patients (5.6%) died and all of them were male (P = 0.075). More details of findings are provided in Table 1.

**TABLE 1:** Demographic and baseline characteristics of patients.

Variable	Result
Age categories	
Under 10	17 (31.5%)
10–14	22 (40.7%)
15 and over	15 (27.8%)
Sex	
Female	27 (50%)
Male	27 (50%)
GI symptoms	
Epigastric pain	38 (70.4%)
Heartburn	1 (1.9%)
Epigastric pain and nausea	9 (16.7%)
Nausea	8 (16.7%)
Epigastric pain and flatulence	1 (1.9%)
Epigastric pan and heart burn	1 (1.9%)
None	4 (7.4%)
Final status	
Alive	51 (94.4%)
Dead	3 (5.6%)

All patients underwent the endoscopic study, and in case of positive findings stool examinations were conducted alongside to ascertain the presence of Helicobacter antigen. According to the endoscopic research, 47 (87%) had symptoms of gastritis, 4 (7.4%) patients presented with both gastritis and esophagitis, and only three patients were reported positive for *H. pylori* infection, all were female and the other 24 females had negative *H. pylori* results. All 27 males had negative Helicobacter study results (P = 0.075). It is noticeable that none of our patients had positive stool antigens. Additionally, those who died did not have severe gastrointestinal symptoms.

The primary etiology of CKD and ultimately end‐stage renal disease (ESRD) was neurogenic bladder (18.5%) and nephronophthisis was identified as an additional etiological factor, observed in 13.06% of cases.

Based on the findings derived from endoscopy, gastritis had been diagnosed in 47 patients (87%) while simultaneous gastritis and esophagitis were identified in 4 cases (7.4%). Generally, famotidine is the most frequently administered drug in dyspepsia 57.4% and the Protein Pump Inhibitors have a market score of 13%. Also, according to medical prescriptions, the pairing of Famotidine and PPI is the most prevalent combination therapy (24.1%) (Table 2). The distribution of GI symptoms and causes of disease according to the Helicobacter status showed no significant difference between the groups (Tables 3 and 4).

**TABLE 2:** Clinical characteristics of patients.

Variable	Result
Pathology results	
Positive	3 (5.6%)
Negative	51 (94.4%)
Endoscopy results	
Gastric	47 (87.0%)
None	3 (5.6%)
Gastric and esophagitis	4 (7.4%)
Cause of the disease	
Systemic lupus erythematosus (SLE)	2 (3.7%)
Cakut (congenital anomalies of the kidney and urinary tract)	1 (1.9%)
Neurogenic bladder	10 (18.5%)
Nephronophthisis	7 (13.06%)
Autosomal recessive polycystic kidney disease (ARPKD)	6 (11.4%)
Focal segmental glomerular sclerosis (FSGS)	6 (11.4%)
Posterior urethral valves (PUV)	5 (9.6%)
Tubulointerstitial nephritis (TIN)	4 (7.6%)
Cystinosis	4 (18.5%)
Hyperoxaluria	4 (18.5%)
Unknown	4 (18.5%)
Renal dysplasia	1 (1.9%)
Endoscopy results	
Gastric	47 (87%)
None	3 (5.6%)
Gastritis and esophagitis	4 (7.4%)
Type of treatment	
Famotidine	31 (57.4%)
Protein pump inhibitors (PPI)	7 (13%)
Famotidine and PPI	13 (24.1%)
Famotidine and antibiotic	1 (1.9%)
PPI and antibiotic	2 (3.7%)

**TABLE 3:** The pathology results according to GI Symptoms.

GI symptoms	Pathology results		Total
	Positive	Negative	
Epigastric pain	3 (5.6%)	35 (68.6%)	38 (70.4%)
None	0 (0.0%)	4 (7.8%)	4 (7.4%)
Heartburn	0 (0.0%)	1 (2.0%)	1 (1.9%)
Epigastric pain and nausea	0 (0.0%)	9 (17.6%)	9 (16.7%)
Epigastric pain and flatulence	0 (0.0%)	1 (2.0%)	1 (1.9%)
Epigastric pan and heart burn	0 (0.0%)	1 (2.0%)	1 (1.9%)
Total	3 (5.6%)	51 (100%)	54 (100%)

**TABLE 4:** The pathology results according to the cause of the disease.

The cause of the disease	Pathology results		Total
	Positive	Negative	
TIN	0 (0.0%)	4 (7.8%)	4 (7.4%)
Cystinosis	0 (0.0%)	4 (7.8%)	4 (7.4%)
NPHP	0 (0.0%)	7 (13.7%)	7 (13.0%)
ARPKD	1 (33.3%)	6 (11.8%)	7 (13.0%)
SLE	1 (33.3%)	1 (2.0%)	2 (3.7%)
FSGS	1 (33.3%)	5 (9.8%)	6 (11.1%)
PUV	0 (0.0%)	5 (9.8%)	5 (9.3%)
Renal dysplasia	0 (0.0%)	1 (2.0%)	1 (1.9%)
Hyperoxaluria	0 (0.0%	4 (7.8%	4 (7.4%)
Neurogenic bladder	0 (0.0%	9 (17.6%)	9 (16.7%
Unknown	0 (0.0%)	4 (7.8%)	4 (7.4%
Pan	0 (0.0%)	1 (2.0%)	1 (1.9%)
Total	3 (100%)	51 (100%)	54 (100%)

## 4 DISCUSSION

CKD is defined as the presence of kidney damage and the loss of kidney function.^12^ One of the symptoms of chronic kidney failure is digestive symptoms, and these patients often complain of indigestion for various reasons. It is considered that uremic toxins and uremic neuropathy, gastrointestinal hypomotility, visceral hypersensitivity to gastric distension, amyloid protein deposition, and *H. pylori* infection have important roles in the development of dyspeptic symptoms in CKD patients. These symptoms are similar to *H. pylori* infection and *H. pylori* infection is also seen in CKD patients as in normal people. Helicobacter infection should be excluded to diagnose the final cause of digestive symptoms as functional indigestion.^5^

The present study is conducted to investigate the prevalence of *H. pylori* in pediatric kidney failure and its relationship with gastrointestinal symptoms. Our results showed there is no significant relationship between the increased risk of chronic kidney failure and *H. pylori* infection and vice versa. Based on our findings the most common gastrointestinal symptom in pediatric CKD is epigastric pain (70.4%).

Increasing interest in assessing the association between *H. pylori* infection and CKD is due to the presence of extraintestinal associations with *H. pylori* infection such as insulin resistance or metabolic syndrome also seen in CKD patients. It is suggested there is a significant relationship between *H. pylori* infection and chronic kidney patients because this infection is more common in those with diabetes and high blood pressure which are the most common causes of CKS.^13^

Based on our findings, there was no notable relationship between *H. pylori* and chronic kidney failure. Similar to our findings, Gu et al.'s study showed no significant association between dialysis‐related chronic renal failure and *H. pylori* infection.^4^ Contrary to our results, Wijarnpreecha et al. stated a significant relationship between chronic renal failure not related to dialysis and *H. pylori* infection.^8^ The difference in sample size may be the cause of this discrepancy. Alhoufie et al. reported the prevalence of acute *H. pylori* infections in 33.1% of kidney failure patients.^14^ In a study by Wijarnpreecha et al., the estimated prevalence of *H. pylori* in ESRD patients is 44%.^8^ In our study, *H. pylori* infection was reported in only three patients with 5.6%. In our study, most of the patients were in the range of 10–14 years old, and the sex distribution was equal. The study by Alhoufie et al. showed no remarkable distribution of *H. pylori* infection among different age groups of renal failure patients.^14^ Matini et al. reported patients' complaints of epigastric pain, nausea, vomiting, then abdominal bloating.^15^ In a study by Ardalan et al. a. majority of renal failure patients usually suffer from gastrointestinal complications such as gastric erosions.^16^ The findings of this study are consistent with the current study. The most prevalent underlying cause of kidney failure in our patients was neurogenic bladder. While Xiong et al. noted 32.9% of *H. pylori*‐seropositivity in renal failure patients suffering from hypertension as an underlying disease which concurs with recent observations.^17^ Also, Alhoufie et al. reported 32.9% of *H. pylori*‐seropositivity in renal failure patients suffering from hypertension as an underlying cause of CKD.^14^ Similar to the current study, in the study by Hooman et al. there was no correlation between *H. pylori* infection and gender.^18^ As CKD patients often complain of dyspepsia, *H. pylori* is a probable cause, but the underlying mechanism is not fully understood. The present study can help in better understanding of this pathology in CKD patients This study has several limitations. First, the sample size was small, and second, there was no control group. Another limitation of the study is its retrospective nature and single‐centric base.

## 5 CONCLUSION

Our study shows no significant relationship between *H. pylori* and chronic kidney failure in children. Due to the low prevalence of *H. pylori* in patients with chronic kidney failure and the association of *H. pylori* with gastrointestinal symptoms, it is suggested that endoscopy and examination should be performed only in symptomatic patients. However, since the number of patients studied in this research was small, we suggest conducting more studies with a larger sample size in several dialysis centers on a larger number of patients. There are concerns that *H. pylori* eradication therapy may worsen kidney function in patients with decreased renal function.

## AUTHOR CONTRIBUTIONS

**Behgol Nemati Nezhad:** Conceptualization; data curation; formal analysis; investigation; supervision. **Bahar Allahverdi:** Conceptualization; investigation; project administration. **Farzaneh Motamed:** Data curation; investigation; methodology; project administration. **Shirin Djalalinia:** Data curation; formal analysis; investigation; methodology; project administration. **Fahimeh Askarian:** Conceptualization; data curation; investigation; project administration. **Daryoush Fahimi:** Conceptualization; data curation; investigation; project administration. **Behnaz Bazargani:** Conceptualization; data curation; investigation; project administration. **Arash Abbasi:** Conceptualization; data curation; investigation; project administration. **Mastaneh Moghtaderi:** Conceptualization; data curation; investigation; methodology; project administration.

## FUNDING INFORMATION

No funding.

## CONFLICT OF INTEREST STATEMENT

There is no conflict of interest.

## ETHICS STATEMENT

The method was approved in compliance with scientific and ethical standards and was performed in line with the relevant guidelines and regulations. The Medical Ethics Committee of Tehran University of Medical Science approved this study. All patients were informed and signed the informed consent form. Batch Number: IR.TUMS.CHMC.REC.1400.075 Date: 1400/04/05 (2021/06/26).

## CONSENT

Written informed consent was obtained from the patient to publish this report in accordance with the journal'spatient consent policy.

## Data Availability

All data and findings of this study are available from the corresponding author upon reasonable request.
